# Single Molecule Localization Microscopy of Mammalian Cell Nuclei on the Nanoscale

**DOI:** 10.3389/fgene.2016.00114

**Published:** 2016-06-24

**Authors:** Aleksander Szczurek, Jun Xing, Udo J. Birk, Christoph Cremer

**Affiliations:** ^1^Superresolution Microscopy, Institute of Molecular BiologyMainz, Germany; ^2^Department of Physics, University of MainzMainz, Germany; ^3^Kirchhoff Institute of Physics, University of HeidelbergHeidelberg, Germany; ^4^Institute of Pharmacy and Molecular Biotechnology, University of HeidelbergHeidelberg, Germany

**Keywords:** single molecule localization microscopy, nuclear structure, super-resolution, nucleoli, DNA dyes

## Abstract

Nuclear texture analysis is a well-established method of cellular pathology. It is hampered, however, by the limits of conventional light microscopy (ca. 200 nm). These limits have been overcome by a variety of super-resolution approaches. An especially promising approach to chromatin texture analysis is single molecule localization microscopy (SMLM) as it provides the highest resolution using fluorescent based methods. At the present state of the art, using fixed whole cell samples and standard DNA dyes, a structural resolution of chromatin in the 50–100 nm range is obtained using SMLM. We highlight how the combination of localization microscopy with standard fluorophores opens the avenue to a plethora of studies including the spatial distribution of DNA and associated proteins in eukaryotic cell nuclei with the potential to elucidate the functional organization of chromatin. These views are based on our experience as well as on recently published research in this field.

## Introduction

The linear DNA of the genome provides the information for the production of various types of RNA molecules; besides mRNAs this includes a large variety of small RNA sequences such as silencing RNAs (siRNA), piRNA, or micro RNAs (miRNA), which play an important role in the regulation of gene expression, development, metabolism as well as in essentially any cellular process. The environment of the genome has a decisive influence on these mechanisms, e.g., by modifying the accessibility to the information encoded within the DNA; it is the basis for normal maintenance of the molecular processes within the cell but its alteration may also result in malfunction and ultimately disease: for example, radiation induced mutations in the DNA sequence may result in cancer related aberrant proteins or gene expression changes, and nuclear structure analysis is a widely used method of cancer diagnosis (Zink et al., 2004; Bergmann and Spector, 2014).

Various models have been proposed to account for the predicted correlation of spatial organization of the nuclear genome and gene function. In this contribution, we would like to discuss in particular some microscopically testable consequences of such models. For example, a recently published model suggests two spatially co-aligned, active and inactive nuclear compartments (ANC and INC; Cremer et al., 2015), in which the INC comprises the compact, transcriptionally inactive core of chromatin domain clusters, and the ANC is formed by the transcriptionally active periphery of chromatin domain clusters, called the perichromatin region, and the interchromatin compartment (IC) (Cremer and Cremer, 2001). The IC is connected to nuclear pores and serves nuclear import and export functions. The ANC is the major site of RNA synthesis. It is highly enriched in epigenetic marks for transcriptionally competent chromatin and RNA Polymerase II (RNAPII). Marks for silent chromatin are enriched in the INC.

One of the basic predictions of this model is the typically large heterogeneity of the nuclear DNA distribution, due to the organization in condensed chromatin domain clusters (INC) and much less condensed ANC compartments. To test such predictions, the spatial resolution of conventional light microscopy (limited to about 200 nm in the object plane and ca. 600 nm along the optical axis) is not sufficient; and combinations of fluorescence microscopy techniques may be employed (Spöri et al., 2004; Rossberger et al., 2013). Electron and enhanced resolution light microscopy (for review see Huang et al., 2009; Cremer and Masters, 2013) have been applied to study the chromatin distribution in mammalian cell nuclei on the nanoscale (Rouquette et al., 2010). For example, using single molecule localization microscopy (SMLM) of human cell nuclei containing histones tagged with fluorescent proteins, light microscopic evidence was obtained for a heterogeneous distribution of chromatin on the nanoscale (Gunkel et al., 2009; Bohn et al., 2010; Markaki et al., 2010). However, in these visualization approaches the average density of detected histone positions was relatively low (around 100/μm^2^), therefore limiting the structural resolution obtained (Nieuwenhuizen et al., 2014; Legant et al., 2016).

To overcome this limitation it is necessary to strongly increase the density of single molecule signals detected (Cremer and Birk, 2016). This goal has been achieved by labeling the DNA directly with low molecular weight DNA dyes. So far Localization Microscopy of DNA was used in attempts to ‘nano-image’ isolated DNA *in vitro* [with fluorescent stains YOYO-1 and PicoGreen (Flors et al., 2009; Schoen et al., 2011)], and the density of DNA *in situ* in fixed and live cells [with fluorescent stains TOPRO (Flors, 2010) and PicoGreen (Benke and Manley, 2012)]. Another group of methods developed in our laboratory takes advantage of a process of photoconversion of Hoechst 33258, Hoechst 33342, and DAPI or Vybrant Violet DNA dyes (Żurek-Biesiada et al., 2013, 2014, 2015; Szczurek et al., 2014). These dyes presumably exist in protonated forms whose absorbance and emission spectra are shifted toward longer wavelengths. By applying an excitation in the blue range (e.g., 488 nm) this approach enables imaging of a small fraction of individual protonated (now green-emitting) fluorophores, and a much higher signal density can be obtained (up to 6000/μm^2^). These novel developments provide a test environment for a multitude of studies on cell nuclei e.g., for the predicted heterogeneity of the nuclear chromatin distribution, and in future for functional structural changes of many epigenetic landmarks and their relation to nuclear DNA at highly enhanced optical and structural resolution. As an example, SMLM can be used to study the spatial distribution of nucleolar on the nanoscale.

## Visualizing Functional Nuclear Structure

In the following, we highlight methodological approaches to obtain super-resolution images of mammalian cell nuclei on the nanoscale. We focus on visualization of directly labeled DNA, and how such approaches can contribute to a super-resolution analysis e.g., of the architecture of ischemic cells or of nucleoli.

### Chromatin Imaging with Directly Labeled DNA

**FIGURE 1:**
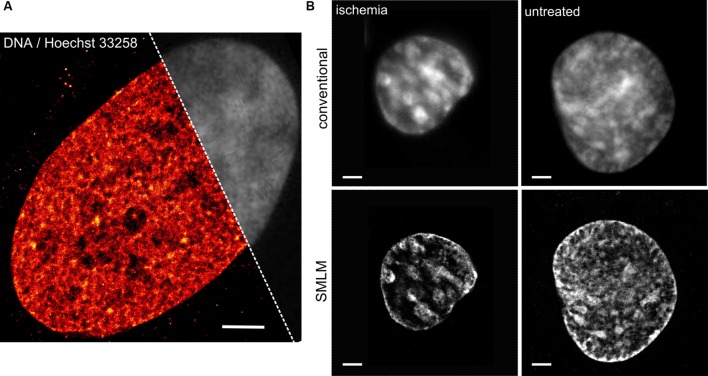
**Direct DNA imaging using localization microscopy. (A)** DNA distribution in an optical section (~600 nm thickness) of a human fibroblast cell nucleus imaged by means of localization microscopy of the Hoechst 33258 photoproduct. The image was acquired with high intensity laser excitation 491 nm combined with low intensity 405 nm light. Approximately 800,000 single Hoechst molecule signals were detected. The individual positions were blurred with the respective localization precision. Partial conventional image presented in gray. Scale bar equals to 2 μm. For details see (Szczurek et al., 2014). **(B)** Chromatin change in HL-1 myocardiac cells upon ischemic conditions studied utilizing single molecule localization microscopy (SMLM) of a standard fluorophore. Cells were incubated for 24 h with EdU–DNA base analog that once incorporated into the DNA during replication was labeled with Alexa 488. The single molecule localization acquisition was performed in a presence of cysteamine serving as a switching agent for Alexa 488. Originally this methodology of super-resolution DNA imaging using EdU–DNA base analog was described by Zessin et al. (2012).

**Figure 1A** presents an SMLM image of the DNA distribution in an optical section (thickness about 600 nm) of a human fibroblast cell nucleus using the DNA dye Hoechst 33258. The image was acquired with high intensity laser excitation 491 nm combined with low intensity 405 nm light. Approximately 800,000 single Hoechst molecule signals were detected. The individual positions were blurred with the respective localization precision. A partial conventional image (upper right) is presented in gray. While the conventional resolution image (presented in gray) indicates some small variations in the DNA density, the super-resolved localization microscopy image (colored in red) clearly indicates differences in DNA density across the nucleus.

Realization of the high resolution imaging of the directly labeled DNA was also proposed utilizing the DNA base analog 5-ethynyl-2′-deoxyuridine (EdU) incorporated into the DNA throughout the entire DNA replication course. Such a treatment followed by a fixation and a click-it reaction of any fluorophore of choice with the exposed chemical group attached to the incorporated bases within the DNA was demonstrated to provide a resolution superior to conventional methods (Zessin et al., 2012). In our hands it provided very similar results to the DNA labeling with e.g., Vybrant Violet. The ease of using typical switchable fluorophores and switching buffers is a huge advantage (Heilemann et al., 2009); however, the adverse biological effects on the cell viability prior to fixation as well as the inability of applying a click chemistry approach for analysis of isolated fixed cell specimens from patients may be considered as a limitation. In such cases, a direct staining with a standard DNA dye, such as presented e.g., in **Figure 1**, may be advantageous.

### Nanoscale Nuclear Architecture of Ischemic Cells

Chromatin compaction status is believed to reflect its local functionality and is likely a cause and a result of processing the DNA information by the local molecular machinery assembled to fulfill a specific role (e.g., replication factories or epigenetic environment just as histone modifications together with associated interacting proteins; Smeets et al., 2014). Thus, SMLM can be considered as a new approach to investigate these processes.

Recently we linked the condensation state of the chromatin studied by means of SMLM of directly labeled DNA to the transcriptional output estimated using standard biochemical methods (Kirmes et al., 2015). We found that the chromatin becomes strongly condensed upon treatment with ischemic conditions i.e., when oxygen and nutrients are deprived. Simultaneously, the transcription level becomes reduced almost by an order of magnitude. In addition, we noticed that the accessibility of antibodies directed against H3 core histones undergoes a considerable decrease. Localization microscopy of directly labeled DNA revealed also that the changes to the status of chromatin are largely reversible and persist only for few tens of minutes after normal conditions have been restituted.

These results of SMLM analysis of chromatin structure and structural changes already were consolidated by more established methods to measure change in DNA digestion rate, light scattering and fluorescence recovery after photobleaching of linker histone H1.1-GFP. All these methods verified the SMLM based conclusions on chromatin redistribution upon ischemia (Kirmes et al., 2015).

### Super-Resolution Imaging of Nucleoli

The eukaryotic cell nucleus comprises several distinct subcompartments of different structure and functionality. An example of such a compartment is the nucleolus: a nucleolar substructure primarily devoid of chromatin, however, bearing an extremely important function in ribosome biogenesis including generation of ribosomal key subunits. Transmission electron microscopy (TEM) emerged as a main method for structural investigation of the nucleolus. Three major structures within the nucleolus have been identified: lightly stained fibrillar centers (FC) constitute about 2% of a typical eukaryotic nucleolus and are surrounded by a dense fibrillar component (DFC) which constitutes approximately 17% of the volume and granular component occupies nearly 75% of the nucleolus (McLeod et al., 2014). Ribosomal DNA (rDNA) transcription units are found in the FCs which consist of tandem repeats of these genes and are harbored by rRNA within the DFC. It is therefore thought that rRNA transcription initiates at the interface between the FC and the DFC.

**FIGURE 2:**
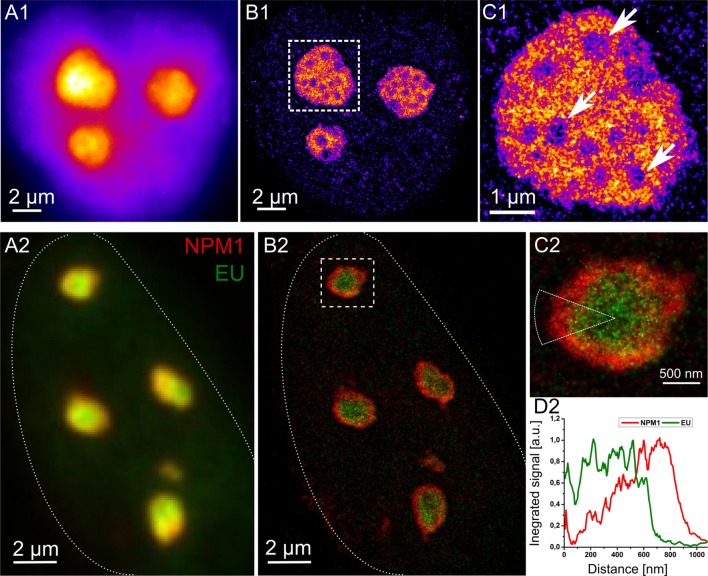
**Super-resolution imaging of cell nucleoli.** Top: Pseudo-color images of a HeLa cell incubated with RNA base analog for 2 h to label nascent RNA transcripts. The cell imaged with conventional widefield microscope **(A1)** and super-resolution reconstruction based on positions of single molecule fluorophores **(B1)**. **(C1)** Demonstrates one of the nucleoli at closer look with new structural details unraveled. Arrows point at the likely positions of fibrillar centers (FC). Bottom: U2OS cell with immunofluorescently labeled NPM1 protein (red) and nascent RNA transcripts stained by means of click chemistry (green) in widefield **(A2)** and in super-resolution **(B2)**. The outline of the cell is indicated with dotted line. **(C2)** Depicts one of the nucleoli where signal of NPM1 and EU was radially integrated within the indicated ROI. **(D2)** Highlights the arrangement of NPM1 and nascent transcripts at the periphery of the nucleolus studied based on single positions of fluorophores. The NPM1 appears as a ring surrounding the nucleolus whereas nascent transcripts occupy rather the central part.

A number of fluorescence labeling techniques including immunofluorescence, nanobodies, click-chemistry, SNAP-technology (Crivat and Taraska, 2012) and many others are available to study nucleolar compounds, e.g., nascent RNA within the nucleolus. RNA synthesis in U2OS cells was fluorescently targeted using the base analog *5-ethynyl uridine* (EU) for 2 h prior to fixation and EU was subsequently conjugated with Alexa 488 switchable fluorophores. The reconstruction of the SMLM fluorophore position map exhibits strong heterogeneity in the distribution of nascent transcripts within the cell nucleolus (**Figure 2** upper part). The signal density attributable to the nascent RNA within the nucleoli varied locally by a factor of 3–4. These features indicate a number of specific subcompartments within the nucleolus and may be used to extract their respective positions: the areas within the nucleolus lacking strong signals (arrows in **Figure 2C1**) probably constitute regions not associated with RNA synthesis, such as the FCs.

Further mapping of the positions of a number of proteins residing in the nucleolus is necessary to elucidate the function of its subcompartmentalization. Such structural analyses can only be performed when high resolution data is available. In **Figure 2**, bottom part, nucleophosmin 1 (NPM1) - a structural protein responsible for i.e., nucleic acid binding, chaperoning, and proliferation (Frehlick et al., 2007), has been imaged together with nascent RNA (EU). This dual color experiment reveals that the nascent RNA is confined mainly to the core part of the nucleoli, whereas NPM1 is directed to the boundary forming there a dense concentration of signal. Similar images elucidating the relative positioning of key proteins in future studies may enable evaluation of the effects of metabolic and/or environmental changes (e.g., oxidative stress, radiation, etc.); or the analysis of diseases on highly ordered protein arrangements and thus might reveal its role in a specific cellular process.

## Discussion: Perspectives for Nuclear Nanoscale Analysis

The functional organization of the nuclear genome still poses an essential challenge to biological research; it also forms a basis for new approaches to health research. Super-resolution microscopy approaches in combination with appropriate labeling techniques now allow us to study chromatin and nuclear texture in unprecedented nanostructural detail, down to the single molecule level. It is anticipated that this will strongly improve the predictive value of such texture analyses. Beyond that, it may be speculated that these techniques might contribute to even larger benefits. For example, would it become possible to alter the compaction of “cancer genes” by specific pharmaceutical molecules, then this might offer the starting point of a new avenue for cancer therapy; another example would be the development of new drugs for the treatment of myocardial infarction and stroke: functional nuclear nanostructure analysis might contribute to test the effects of drugs on alteration to the drastic changes induced by oxygen and nutrient deficiency in chromatin nanostructure (and therefore in gene activity). Today, such speculations may still appear as “science fiction”; however, a thorough understanding of the architecture of the cell nucleus on the nanoscale, might provide an essential step toward its realization.

The growing number of publications reporting on super-resolution analysis of mammalian cell nuclei highlights the increasing interest in the interplay between chromatin structure and regulatory mechanisms. Recently, an example of nanostructure analysis in stem cells relying on localization microscopy-imaging of labeled core histones imaged at different pluripotency stages was published (Ricci et al., 2015). Using sophisticated quantitative analysis tools, the authors of this study showed that histone-targeting single molecule signals form specific ‘clutches’ the size of which decreased with an increasing pluripotency.

Although the global nuclear nanostructure assessment using SMLM is of great importance, the goal remains to visualize and hence quantitatively analyze in high-resolution the chromatin status of individual gene loci. This has been demonstrated using SMLM by means of conventional *in situ* hybridization (Weiland et al., 2011), or by using specific single oligonucleotides (Stuhlmüller et al., 2015). Most recently this has been also demonstrated using oligo-paint DNA probes that yield a signal accumulation only upon transient binding to the sequence of interest. In this case a library of direct FISH probes was designed and each probe contained a common overhang sequence responsible for interacting with oligo-paint probes (Beliveau et al., 2015). Using such techniques, an unprecedented specificity of labeling isolated gene loci in 3D intact nuclei was demonstrated. This approach may provide complementary information on particular genes to the data obtained using next generation sequencing approaches and chromosomal conformation capture (Dekker et al., 2002, 2013; van Berkum et al., 2010).

All aforementioned localization microscopy images were obtained with microscopes in widefield illumination mode. However, different illumination schemes are also available e.g., using light sheet illumination in combination with single molecule detection, effectively suppressing out-of-focus background, yielding high quality single molecule localization signals originating from only one section through the nucleus. By doing so, it was possible to study the spatial organization of RNAPII as well as to quantify its clustering (Zhao et al., 2014). This study revealed that 70% of these ‘clusters’ originated from single RNAPII molecules, arguing against the simultaneous recruitment of most of RNAPII molecules into transcription factories. In another study, the authors set out to investigate Sox2 enhancer organization in living embryonic cells using light-sheet illumination along with localization microscopy imaging using Fluorescent Proteins (Liu et al., 2014). They demonstrated that Sox2 transcription factor targets a subset of RNAPII-enriched regions in the nucleus and hypothesized that this encourages the gene to undergo transcription. These pioneering studies highlight the importance of further technological developments.

With the advent of localization based super-resolution microscopy of nuclear DNA using standard dyes, the enhanced structural resolution allows the heterogeneity of nuclear compartments to be categorized into further substructures the function of which may be elucidated. SMLM microscopy techniques are highly useful to study these nanostructures down to single molecule resolution. This proves the versatility and usefulness of the novel super-resolution methods in combination with advanced labeling (and embedding) approaches in further structural studies of the cell nucleus. Fields of application envisaged include among others DNA repair, genome instability, development and differentiation, epigenetics in the context of nuclear structure, neuromedicine (e.g., neurological disorders).

## Author Contributions

UB, CC, and AS drafted the manuscript; AS and JX performed experiments and data evaluation; UB and AS worked on the microscope setup.

## Conflict of Interest Statement

The authors declare that the research was conducted in the absence of any commercial or financial relationships that could be construed as a potential conflict of interest. The reviewer MS and handling Editor declared their shared affiliation, and the handling Editor states that the process nevertheless met the standards of a fair and objective review.
